# ﻿Morphological and phylogenetic analyses reveal two new species and a new record of *Apiospora* (Amphisphaeriales, Apiosporaceae) in China

**DOI:** 10.3897/mycokeys.95.96400

**Published:** 2023-01-27

**Authors:** Rongyu Liu, Duhua Li, Zhaoxue Zhang, Shubin Liu, Xinye Liu, Yixin Wang, Heng Zhao, Xiaoyong Liu, Xiuguo Zhang, Jiwen Xia, Yujiao Wang

**Affiliations:** 1 College of Life Sciences, Shandong Normal University, Jinan, 250358, China Shandong Normal University Jinan China; 2 Shandong Provincial Key Laboratory for Biology of Vegetable Diseases and Insect Pests, College of Plant Protection, Shandong Agricultural University, Taian, 271018, China Shandong Agricultural University Taian China; 3 Institute of Microbiology, School of Ecology and Nature Conservation, Beijing Forestry University, Beijing, 100083, China Beijing Forestry University Beijing China

**Keywords:** *Apiosporadongyingensis*, *Apiosporahainanensis*, Ascomycota, bamboo, taxonomy

## Abstract

The genus *Apiospora* includes endophytes, pathogens and saprobes, with a wide host range and geographic distribution. In this paper, six *Apiospora* strains isolated from diseased and healthy tissues of bamboo leaves from Hainan and Shandong provinces in China were classified using a multi-locus phylogeny based on a combined dataset of ITS, LSU, *tef1* and *tub2*, in conjunction with morphological characters, host association and ecological distribution. Two new species, *Apiosporadongyingensis* and *A.hainanensis*, and a new record of *A.pseudosinensis* in China, are described based on their distinct phylogenetic relationships and morphological analyses. Illustrations and descriptions of the three taxa are provided, along with comparisons with closely related taxa in the genus.

## ﻿Introduction

*Apiospora* Sacc., the type genus of Apiosporaceae K.D. Hyde, J. Fröhl., Joanne E. Taylor & M.E. Barr, was introduced by Saccardo with *A.montagnei* Sacc. as the type species (Saccardo 1875). The sexual morphs of *Apiospora* are characterized by multi-locular perithecial stromata with hyaline ascospores surrounded by a thick gelatinous sheath (Dai et al. 2016, 2017; Pintos and Alvarado 2021). The asexual morphs of *Apiospora* are characterized by their basauxic conidiogenesis, and globose to subglobose conidia, which are usually lenticular or obovoid in the side view, and pale brown to brown in color (Kunze 1817; Hyde et al. 1998; Dai et al. 2016). Most species of *Apiospora* are quite similar to each other in morphology, thus it is difficult to distinguish them without molecular phylogenetic data.

Until the studies of Pintos and Alvarado (2021) and Jiang et al. (2022a), the closely related genera *Apiospora*, *Arthrinium* Kunze and *Neoarthrinium* Ning Jiang were considered a single taxon because of their similar morphological characteristics, especially the basauxic conidiogenesis. However, the conidia of *Apiospora* and *Neoarthrinium* are more or less rounded in the face view and lenticular in the side view, whereas the conidia of *Arthrinium* are variously shaped (angular, curved, fusiform, globose, polygonal, navicular). In addition, the conidiophores of several *Arthrinium* and *Neoarthrinium* species have thick blackish septa, which are rarely observed in *Apiospora* (Pintos and Alvarado 2021; Tian et al. 2021; Jiang et al. 2022a). *Apiospora* species have a worldwide distribution and can be found on various hosts, while *Arthrinium* species are rarely found in tropical and subtropical habitats and commonly occur on Cyperaceae Juss. and Juncaceae Juss. (Ramos et al. 2010; Dai et al. 2017; Wang et al. 2018; Hyde et al. 2020; Pintos and Alvarado 2021; Tian et al. 2021). Four *Neoarthrinium* species have been discovered on four hosts from three distantly related host plant families in China, Colombia and Great Britain (Jiang et al. 2022a). Most *Apiospora* species are associated with plants as endophytes, pathogens or saprobes (Agut and Calvo 2004; Dai et al. 2016, 2017; Tian et al. 2021). Some species are economically important plant pathogens, for example, *A.arundinis* causes bamboo brown culm streak, chestnut leaf spot and barley kernel blight (Martínez-Cano et al. 1992; Chen et al. 2014; Jiang et al. 2021), while *A.sacchari* causes damping-off of durum wheat (Mavragani et al. 2007). Some species have also been isolated from lichens, air, soil, seaweeds and animal tissues, and a few species are human pathogens which can cause cutaneous infections (Tian et al. 2021).

The aim of this study was to explore the diversity of *Apiospora* species in symptomatic and asymptomatic bamboo leaves collected in Hainan and Shandong provinces (China). We describe two newly discovered species, *Apiosporadongyingensis* and *A.hainanensis*, and a new record of *A.pseudosinensis* in China based on phylogenetic data and morphology.

## ﻿Materials and methods

### ﻿Isolation and morphological studies

The samples were collected at the Diaoluoshan National Nature Reserve, Hainan Province, and the Dongying Botanical Garden, Shandong Province (China). The strains of *Apiospora* were isolated from single spores and fungal tissue obtained from diseased and healthy bamboo leaves following the methods described by Chomnunti et al. (2014). Sampled spores were suspended in sterile distilled water, spread onto potato dextrose agar (PDA) plates, and incubated for one day at 25 °C. After germination, the spores were transferred to a new PDA plate to obtain a pure culture. Additionally, about 25 mm^2^ tissue fragments were taken from the margin of leaf lesions and their surface sterilized by consecutive immersions in a 75% ethanol solution for 60 s, 5% sodium hypochlorite solution for 30 s, and then rinsed in sterile distilled water for 60 s (Mu et al. 2021). The surface sterilized plant tissue was dried with sterilized paper and moved on the PDA plates. All the PDA plates were incubated at 25 °C for 3–4 days in darkness, and then hyphae were picked out of the periphery of the colonies and grown on new PDA plates (Jiang et al. 2022b).

After 7 days of incubation, the morphological characters of the colonies were recorded on PDA with a digital camera (Canon G7X). Morphological descriptions were based on cultures sporulating on water agar (WA). The size of the conidiogenous cells and conidia were shown as minimum-maximum. Color notations were done using the color charts of Rayner (1970). The micro-morphological characters of the colonies were studied using a stereomicroscope (Olympus SZX10) and a microscope (Olympus BX53), both fitted with high-definition color digital cameras. Grown cultures of *Apiospora* were stored in 10% sterilized glycerin and sterile water at 4 °C for further studies in the future. All specimens were deposited in the Herbarium of the Department of Plant Pathology, Shandong Agricultural University (**HSAUP**). Living cultures were deposited in the Shandong Agricultural University Culture Collection (**SAUCC**). Taxonomic information on the new taxa was submitted to MycoBank (http://www.mycobank.org).

### ﻿DNA extraction and amplification

Genomic DNA was extracted from fungal mycelia grown on PDA, using a modified cetyltrimethylammonium bromide (CTAB) protocol as described in Guo et al. (2000). DNA sequences of four different loci were obtained, including the nrDNA internal transcribed spacer regions 1 and 2 with the intervening 5.8S subunit (ITS), a partial sequence of the large subunit nrDNA subunit (LSU), a partial sequence of the translation elongation factor 1-alpha gene (*tef1*), and a partial sequence of the beta-tubulin gene (*tub2*). They were all amplified with the primer pairs and polymerase chain reaction (PCR) program listed in Table 1.

**Table 1.** Gene regions and respective primer pairs used in the study.

Locus	PCR primers	PCR: thermal cycles: (Annealing temperature in bold)	Reference
ITS	ITS5/ITS4	(94 °C: 30 s, **55 °C**: 30 s, 72 °C: 45 s) × 29 cycles	White et al. 1990
LSU	LR0R/LR5	(94 °C: 30 s, **48 °C**: 50 s, 72 °C: 1 min 30 s) × 35 cycles	Vilgalys and Hester 1990; Cubeta et al. 1991
*tef1*	EF1-728F/EF2	(95 °C: 30 s, **51 °C**: 30 s, 72 °C: 1 min) × 35 cycles	O’Donnell et al. 1998; Carbone and Kohn 1999
*tub2*	Bt-2a/Bt-2b	(95 °C: 30 s, **56 °C**: 30 s, 72 °C: 1 min) × 35 cycles	Glass and Donaldson 1995

PCR was performed using an Eppendorf Master Thermocycler (Hamburg, Germany). Amplification reactions contained 12.5 μL 2× Taq Plus Master Mix II (Vazyme, Nanjing, China), 1 μL of each forward and reverse primers (10 μM) (Tsingke, Qingdao, China), 1 μL of template genomic DNA, and distilled deionized water to a total volume of 25 μL. The PCR products were visualized on 1% agarose electrophoresis gels. Bi-directional sequencing was conducted by the Tsingke Company Limited (Qingdao, China). Consensus sequences were obtained using MEGA 7.0 (Kumar et al. 2016). All sequences generated in this study were deposited in GenBank (Table 2).

**Table 2.** Isolates and GenBank accession numbers used in the phylogenetic analyses.

Species	Isolate/Strain	Host/Substrate	Origin	GenBank accession numbers			
				ITS	LSU	*tef1*	*tub2*
*Apiosporaacutiapica*	KUMCC 20-0210 (Type)	*Bambusabambos*	China	MT946343	MT946339	MT947360	MT947366
*A.agari*	KUC21333 (Type)	*Agarumcribrosum*	Korea	MH498520	MH498440	MH544663	MH498478
*A.aquatica*	S-642 (Type)	Submerged wood	China	MK828608	MK835806	NA	NA
*A.arctoscopi*	KUC21331 (Type)	Egg of *Arctoscopusjaponicus*	Korea	MH498529	MH498449	MN868918	MH498487
*A.arundinis*	CBS 124788	Living leaves of *Fagussylvatica*	Switzerland	KF144885	KF144929	KF145017	KF144975
*A.aurea*	CBS 244.83 (Type)	Air	Spain	AB220251	KF144935	KF145023	KF144981
*A.balearica*	CBS 145129 (Type)	Undetermined Poaceae	Spain	MK014869	MK014836	MK017946	MK017975
*A.biserialis*	CGMCC 3.20135 (Type)	Bamboo	China	MW481708	MW478885	MW522938	MW522955
*A.camelliae-sinensis*	LC5007 (Type)	*Camelliasinensis*	China	KY494704	KY494780	KY705103	KY705173
*A.chiangraiense*	MFLUCC21-0053 (Type)	Dead culms of bamboo	Thailand	MZ542520	MZ542524	NA	MZ546409
*A.chromolaenae*	MFLUCC 17-1505 (Type)	*Chromolaenaodorata*	Thailand	MT214342	MT214436	NA	NA
*A.cordylines*	GUCC 10027 (Type)	Leaves of *Cordylinefruticosa*	China	MT040106	NA	MT040127	MT040148
*A.cyclobalanopsidis*	CGMCC 3.20136 (Type)	*Cyclobalanopsidisglauca*	China	MW481713	MW478892	MW522945	MW522962
*A.descalsii*	CBS 145130 (Type)	*Ampelodesmosmauritanicus*	Spain	MK014870	MK014837	MK017947	MK017976
*A.dichotomanthi*	LC4950 (Type)	*Dichotomanthustristaniaecarpa*	China	KY494697	KY494773	KY705096	KY705167
***A.dongyingensis***	**SAUCC 0302 (Type)**	**Leaf of bamboo**	**China**	**OP563375**	**OP572424**	**OP573264**	**OP573270**
	**SAUCC 0303**	**Leaf of bamboo**	**China**	**OP563374**	**OP572423**	**OP573263**	**OP573269**
*A.esporlensis*	CBS 145136 (Type)	*Phyllostachysaurea*	Spain	MK014878	MK014845	MK017954	MK017983
*A.euphorbiae*	IMI 285638b	*Bambusa* sp.	Bangladesh	AB220241	AB220335	NA	AB220288
*A.fermenti*	KUC21289 (Type)	Seaweed	Korea	MF615226	MF615213	MH544667	MF615231
*A.gaoyouensis*	CFCC 52301 (Type)	*Phragmitesaustralis*	China	MH197124	NA	MH236793	MH236789
*A.garethjonesii*	JHB004 (Type)	Culms of dead bamboo	China	KY356086	KY356091	NA	NA
*A.gelatinosa*	HKAS 111962 (Type)	Culms of dead bamboo	China	MW481706	MW478888	MW522941	MW522958
*A.guiyangensis*	HKAS 102403 (Type)	Dead culms of Poaceae	China	MW240647	MW240577	MW759535	MW775604
*A.guizhouensis*	LC5322 (Type)	Air in karst cave	China	KY494709	KY494785	KY705108	KY705178
***A.hainanensis***	**SAUCC 1681 (Type)**	**Leaf of bamboo**	**China**	**OP563373**	**OP572422**	**OP573262**	**OP573268**
	**SAUCC 1682**	**Leaf of bamboo**	**China**	**OP563372**	**OP572421**	**OP573261**	**OP573267**
*A.hispanica*	IMI 326877 (Type)	Maritime sand	Spain	AB220242	AB220336	NA	AB220289
*A.hydei*	CBS 114990 (Type)	Culms of *Bambusatuldoides*	China	KF144890	KF144936	KF145024	KF144982
*A.hyphopodii*	MFLUCC 15-0003 (Type)	Dead culms of bamboo	Thailand	KR069110	NA	NA	NA
*A.hysterina*	ICPM 6889 (Type)	Bamboo	New Zealand	MK014874	MK014841	MK017951	MK017980
*A.iberica*	AP10118 (Type)	*Arundodonax*	Portugal	MK014879	MK014846	MK017955	MK017984
*A.intestini*	CBS 135835 (Type)	Gut of grasshopper	India	KR011352	KR149063	KR011351	KR011350
*A.italica*	CBS 145138 (Type)	*Arundodonax*	Italy	MK014880	MK014847	MK017956	MK017985
*A.jatrophae*	CBS 134262 (Type)	*Jatrophapodagrica*	India	JQ246355	NA	NA	NA
*A.jiangxiensis*	LC4577 (Type)	*Maesa* sp.	China	KY494693	KY494769	KY705092	KY705163
*A.kogelbergensis*	CBS 113333 (Type)	Dead culms of *Restionaceae*	South Africa	KF144892	KF144938	KF145026	KF144984
*A.koreana*	KUC21332 (Type)	Egg of *Arctoscopusjaponicus*	Korea	MH498524	MH498444	MH544664	MH498482
*A.locuta-pollinis*	LC11683 (Type)	*Brassicacampestris*	China	MF939595	NA	MF939616	MF939622
*A.longistroma*	MFLUCC 11-0481 (Type)	Culms of decaying bamboo	Thailand	KU940141	KU863129	NA	NA
*A.malaysiana*	CBS 102053 (Type)	*Macarangahullettii* stem colonised by ants	Malaysia	KF144896	KF144942	KF145030	KF144988
*A.marianiae*	AP18219 (Type)	Dead stems of *Phleumpratense*	Spain	ON692406	ON692422	ON677180	ON677186
*A.marii*	CBS 497.90 (Type)	Atmosphere, pharmaceutical excipients, home dust and beach sands	Spain	MH873913	KF144947	KF145035	KF144993
*A.marina*	KUC21328 (Type)	Seaweed	Korea	MH498538	MH498458	MH544669	MH498496
*A.mediterranea*	IMI 326875 (Type)	Air	Spain	AB220243	AB220337	NA	AB220290
*A.minutispora*	17E-042 (Type)	Soil	South Korea	LC517882	NA	LC518889	LC518888
*A.montagnei*	AP301120 (Epitype)	*Arundomicrantha*	Spain	ON692408	ON692424	ON677182	ON677188
	AP19421	*Arundomicrantha*	Spain	ON692418	ON692425	ON677183	ON677189
	CPC 18900	Culms of *Phragmitesaustralis*	Italy	KF144909	KF144956	KF145043	KF145001
*A.mori*	MFLU 18-2514 (Type)	Dead leaves of *Morusaustralis*	China	MW114313	MW114393	NA	NA
*A.multiloculata*	MFLUCC 21-0023 (Type)	Dead culms of *Bambusae*	Thailand	OL873137	OL873138	NA	OL874718
*A.mytilomorpha*	DAOM 214595 (Type)	Dead blades of *Andropogon* sp.	India	KY494685	NA	NA	NA
*A.neobambusae*	LC7106 (Type)	Leaf of bamboo	China	KY494718	KY494794	KY806204	KY705186
*A.neochinense*	CFCC 53036 (Type)	*Fargesiaqinlingensis*	China	MK819291	NA	MK818545	MK818547
*A.neogarethjonesii*	HKAS 102408 (Type)	Dead culms of *Bambusae*	China	MK070897	MK070898	NA	NA
*A.neosubglobosa*	JHB007 (Type)	Bamboo	China	KY356090	KY356095	NA	NA
*A.obovata*	LC4940 (Type)	*Lithocarpus* sp.	China	KY494696	KY494772	KY705095	KY705166
*A.ovata*	CBS 115042 (Type)	*Arundinariahindsii*	China	KF144903	KF144950	KF145037	KF144995
*A.paraphaeosperma*	MFLUCC13-0644 (Type)	Dead clumps of *Bambusa* sp.	Thailand	KX822128	KX822124	NA	NA
*A.phyllostachydis*	MFLUCC 18-1101 (Type)	*Phyllostachysheteroclada*	China	MK351842	MH368077	MK340918	MK291949
*A.piptatheri*	CBS 145149 (Type)	*Piptatherummiliaceum*	Spain	MK014893	MK014860	MK017969	NA
*A.pseudomarii*	GUCC 10228 (Type)	Leaves of *Aristolochiadebilis*	China	MT040124	NA	MT040145	MT040166
*A.pseudoparenchymatica*	LC7234 (Type)	Leaf of bamboo	China	KY494743	KY494819	KY705139	KY705211
*A.pseudorasikravindrae*	KUMCC 20-0208 (Type)	*Bambusadolichoclada*	China	MT946344	NA	MT947361	MT947367
*A.pseudosinensis*	CPC 21546 (Type)	Leaf of bamboo	Netherlands	KF144910	KF144957	KF145044	MN868936
***A.pseudosinensis***	**SAUCC 0221**	**Leaf of bamboo**	**China**	**OP563377**	**OP572426**	**OP573266**	**OP573272**
	**SAUCC 0222**	**Leaf of bamboo**	**China**	**OP563376**	**OP572425**	**OP573265**	**OP573271**
*A.pseudospegazzinii*	CBS 102052 (Type)	*Macarangahullettii* stem colonized by ants	Malaysia	KF144911	KF144958	KF145045	KF145002
*A.pterosperma*	CPC 20193 (Type)	*Lepidospermagladiatum*	Australia	KF144913	KF144960	KF145046	KF145004
*A.pusillisperma*	KUC21321 (Type)	Seaweed	Korea	MH498533	MH498453	MN868930	MH498491
*A.qinlingensis*	CFCC 52303 (Type)	*Fargesiaqinlingensis*	China	MH197120	NA	MH236795	MH236791
*A.rasikravindrae*	LC5449	Soil in karst cave	China	KY494713	KY494789	KY705112	KY705182
*A.sacchari*	CBS 212.30	*Phragmitesaustralis*	UK	KF144916	KF144962	KF145047	KF145005
*A.saccharicola*	CBS191.73	Air	Netherlands	KF144920	KF144966	KF145051	KF145009
*A.sargassi*	KUC21228 (Type)	*Sargassumfulvellum*	Korea	KT207746	KT207696	MH544677	KT207644
*A.sasae*	CBS 146808 (Type)	Dead culms of *Sasaveitchii*	Netherlands	MW883402	MW883797	MW890104	MW890120
*A.septata*	CGMCC 3.20134 (Type)	Bamboo	China	MW481711	MW478890	MW522943	MW522960
*A.serenensis*	IMI 326869 (Type)	Food, pharmaceutical excipients, atmosphere and home dust	Spain	AB220250	AB220344	NA	AB220297
*A.setariae*	CFCC 54041 (Type)	Decaying culms of *Setariaviridis*	China	MT492004	NA	NA	NA
*A.sichuanensis*	HKAS 107008 (Type)	Dead culms of Poaceae	China	MW240648	MW240578	MW759536	MW775605
*A.sorghi*	URM 93000 (Type)	*Sorghumbicolor*	Brazil	MK371706	NA	NA	MK348526
*A.sphaerosperma*	CBS114314	Leaf of *Hordeumvulgare*	Iran	KF144904	KF144951	KF145038	KF144996
*A.stipae*	CBS 146804 (Type)	Dead culm of *Stipagigantea*	Spain	MW883403	MW883798	MW890082	MW890121
*A.subglobosa*	MFLUCC 11-0397 (Type)	Dead bamboo culms	Thailand	KR069112	KR069113	NA	NA
*A.subrosea*	LC7292 (Type)	Leaf of bamboo	China	KY494752	KY494828	KY705148	KY705220
*A.thailandica*	LC5630	Rotten wood	China	KY494714	KF144970	KY705113	KY806200
*A.vietnamensis*	IMI 99670 (Type)	*Citrussinensis*	Vietnam	KX986096	KX986111	NA	KY019466
*A.xenocordella*	CBS 478.86 (Type)	Soil from roadway	Zimbabwe	KF144925	KF144970	KF145055	KF145013
*A.yunnana*	MFLUCC 15-0002 (Type)	Decaying bamboo culms	China	KU940147	KU863135	NA	NA
*Arthriniumcaricicola*	CBS 145127	*Carexericetorum*	China	MK014871	MK014838	MK017948	MK017977

Notes: Strains in this study are marked in bold. NA = not available.

### ﻿Phylogenetic analyses

Newly generated ITS, LSU, *tef1* and *tub2* sequences from the six strains studied were aligned with all reference sequences of *Apiospora* and related species available in GenBank using the MAFFT v.7.11 online software (http://mafft.cbrc.jp/alignment/server/, Katoh et al. 2019) with the default settings, manually correcting the resulting alignment where necessary. Maximum likelihood (ML) and Bayesian inference (BI) phylogenetic analyses were conducted individually on each locus (ITS, LSU, *tef1* and *tub2*) and on a combined dataset including all of them. The best-fitting evolutionary model of each partition was determined using MrModeltest v. 2.3 (Nylander 2004). ML and BI were run on the CIPRES Science Gateway portal (https://www.phylo.org/) using RaxML-HPC2 on XSEDE (8.2.12) (Miller et al. 2012; Stamatakis 2014) and MrBayes on XSEDE (3.2.7a), respectively (Huelsenbeck and Ronquist 2001; Ronquist and Huelsenbeck 2003; Ronquist et al. 2012). For ML analyses the default parameters were used, while BI was carried out using a Markov chain Monte Carlo (MCMC) algorithm. BI analyses included four MCMC chains and were run for 5,000,000 generations until the average standard deviation of split frequencies was below 0.01 with trees saved every 1000 generations. The burn-in fraction was set to 0.25 and posterior probabilities (PP) were determined from the remaining trees. The resulting 50% majority-rule tree was plotted using FigTree v. 1.4.4 (http://tree.bio.ed.ac.uk/software/figtree) and edited with Adobe Illustrator CS6.0.

## ﻿Results

### ﻿Phylogenetic analyses

Among the six strains of *Apiospora* isolated from the samples studied, two new species were discovered, and another one found for the first time in China after the combined analysis of ITS, LSU, *tef1* and *tub2* DNA sequences from 89 isolates of *Apiospora* plus *Arthriniumcaricicola* Kunze & J.C. Schmidt (CBS 145127) as the outgroup taxon.

A total of 2241 characters including gaps were compared in the phylogenetic analysis, viz. ITS: 1–706, LSU: 707–1513, *tef1*: 1514–1932, *tub2*: 1933–2241. Of these characters, 1436 were constant, 271 were variable and parsimony-uninformative, and 534 were parsimony-informative. For the BI and ML analyses, the substitution model GTR+I+G was selected for all partitions.

**Figure 1.**
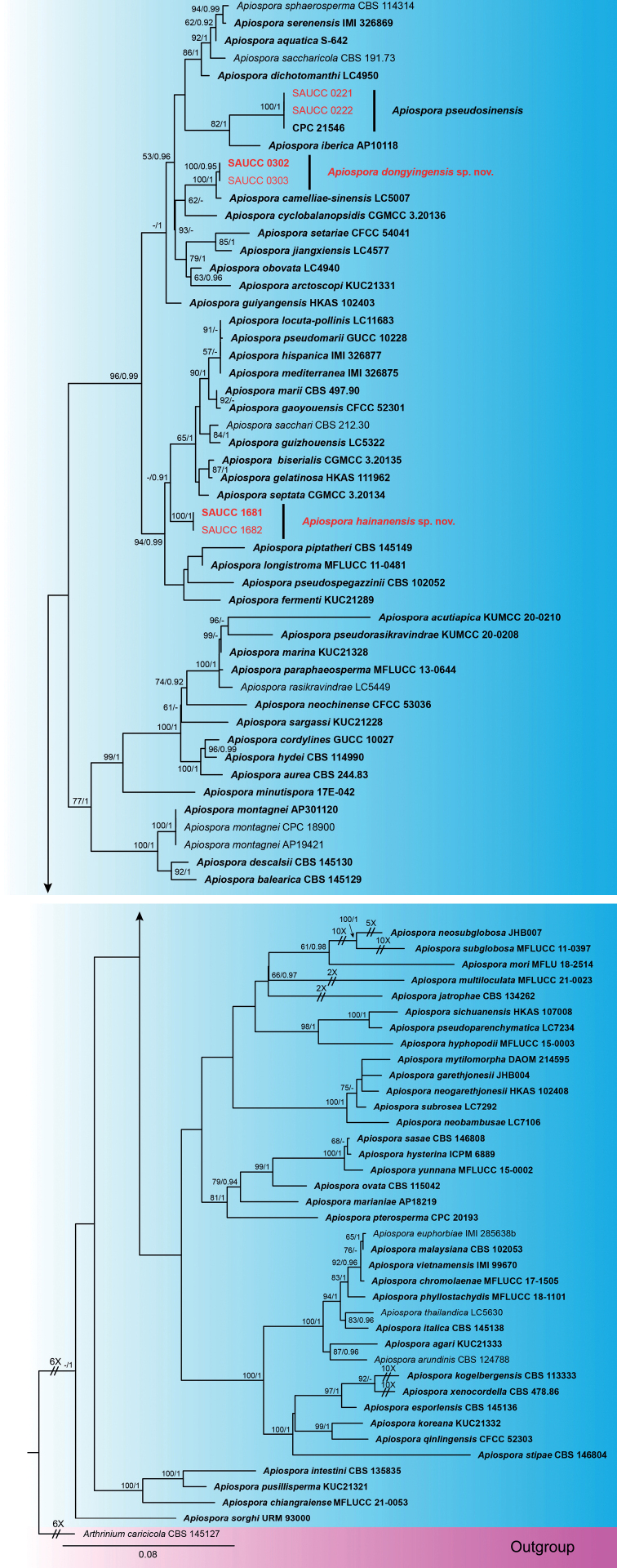
Phylogram of *Apiospora* based on combined ITS, LSU, *tef1* and *tub2* genes. ML bootstrap support values (MLBS ≥ 50%) and Bayesian posterior probability (BYPP ≥ 0.90) are shown as first and second position above nodes, respectively. Strains from this study are shown in red, ex-type or ex-epitype cultures are indicated in bold face. Some branches were shortened according to the indicated mulipliers.

The BI analysis reached the established convergence after 3935000 generations, resulting in 39351 sampled trees, of which 29514 trees were used to calculate the posterior probabilities. The ML tree topology agreed with that obtained from the BI analysis, and therefore, only one tree (the ML) is presented (Fig. 1). The four strains (SAUCC 0302, SAUCC 0303, SAUCC 1681 and SAUCC 1682) studied in the present work represent two independent clades, interpreted as newly discovered independent species. These are described below and accommodated under the new names *Apiosporadongyingensis* and *A.hainanensis*. Another two strains (SAUCC 0221 and SAUCC 0222) clustered with *A.pseudosinensis* (CPC 21546) with full support (MLBS: 100% and BYPP: 1), and are therefore considered no different from this species.

### ﻿Taxonomy

#### ﻿ Apiospora dongyingensis

Taxon classificationFungiXylarialesApiosporaceae

R.Y. Liu, J.W. Xia & X.G. Zhang sp. nov.

414B86CE-7C66-5FA3-928A-9E981DC14E0F

846065

Fig. 2

##### Etymology.

Named after Dongying City (China) where the type was collected.

##### Type.

China, Shandong Province: Dongying Botanical Garden, on diseased leaves of bamboo, 13 July 2022, R.Y. Liu, holotype HSAUP 0302, ex-type living culture SAUCC 0302.

##### Description.

***Asexual morph***: On WA, hyphae 1.3–3.6 μm diam., hyaline, branched, septate. Conidiophores cylindrical, septate, verrucose, flexuous, sometimes reduced to conidiogenous cells. Conidiogenous cells globose to subglobose, erect, blastic, aggregated in clusters on hyphae, hyaline to pale brown, smooth, branched, 8.2–13.9 × 4.2–8.2 μm, mean ± SD: 9.6 ± 1.6 × 6.7 ± 1.1 μm (n = 40). Conidia globose, subglobose to lenticular, with a longitudinal germ slit, occasionally elongated to ellipsoidal, brown to dark brown, smooth to finely roughened, 8.0–16.5 × 5.5–9.0 μm, mean ± SD: 9.4 ± 1.9 × 7.3 ± 1.0 μm, L/W = 1.3–1.9 (n = 40). ***Sexual morph***: Undetermined.

**Figure 2.**
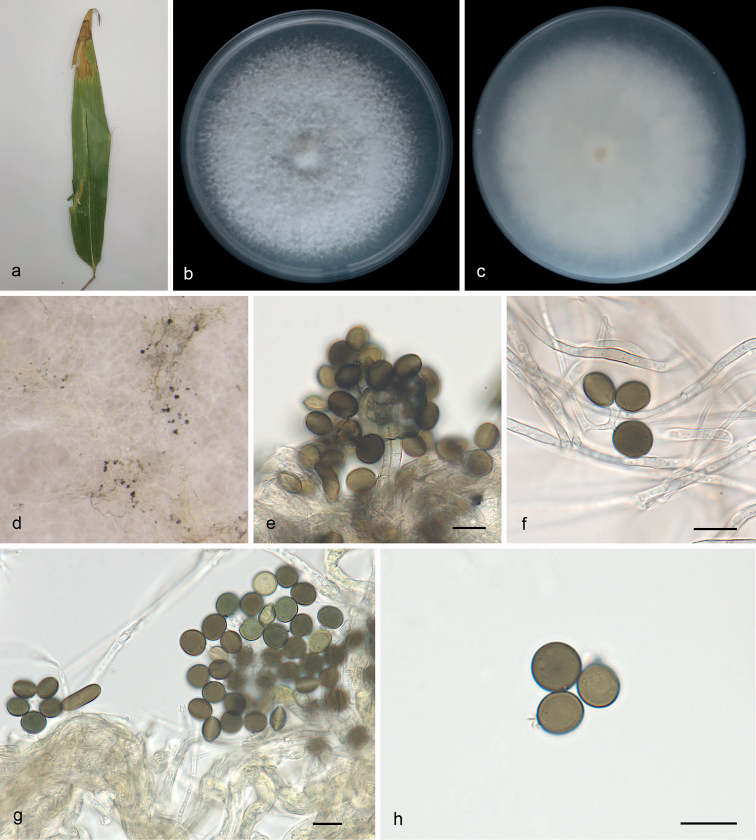
*Apiosporadongyingensis* (SAUCC 0302, ex-holotype culture) **a** leaf of host plant **b, c** surface (**b**) and reverse (**c**) sides of colony after incubation for 7 days on PDA**d** conidiomata formed in culture **e, f** conidiogenous cells and conidia **g, h** conidia. Scale bars: 10 μm (**e–h**).

##### Culture characteristics.

Colonies on PDA flat with entire margin, aerial mycelium white to gray, floccose cottony; surface and reverse gray in the center and grayish margin. PDA attaining 78.5–86.5 mm in diameter after 7 days at 25 °C, growth rate 11.0–12.5 mm/day.

##### Additional specimen examined.

China, Shandong Province: Dongying Botanical Garden, on diseased leaves of bamboo, 13 July 2022, R.Y. Liu, paratype HSAUP 0303, ex-paratype living culture SAUCC 0303.

##### Notes.

*Apiosporadongyingensis* is closely related but phylogenetically distinct from *A.camelliae-sinensis* (M. Wang, F. Liu & L. Cai) Pintos & P. Alvarado and *A.cyclobalanopsidis* (Y. Feng & Jian K. Liu) X.G. Tian & Tibpromma (Fig. 1). *A.dongyingensis* differs from *A.camelliae-sinensis* by 18 nucleotides (13/518 in ITS, 2/804 in LSU, 2/374 in *tef1* and 1/265 in *tub2*) and *A.cyclobalanopsidis* by 58 nucleotides (17/518 in ITS, 4/799 in LSU, 26/377 in *tef1* and 11/266 in *tub2*). Morphologically, it differs from *A.camelliae-sinensis* and *A.cyclobalanopsidis* in its conidia (globose, subglobose to lenticular, 8.0–16.5 × 5.5–9.0 μm in *A.dongyingensis* vs. globose to subglobose, 9.0–13.5 × 7.0–12.0 μm in *A.camelliae-sinensis* and surface view globose to ellipsoid, 8–12 μm long and side view lenticular, 10–14 μm long in *A.cyclobalanopsidis*; Wang et al. 2018; Feng et al. 2021; Pintos and Alvarado 2021; Tian et al. 2021).

#### ﻿ Apiospora hainanensis

Taxon classificationFungiXylarialesApiosporaceae

R.Y. Liu, J.W. Xia & X.G. Zhang sp. nov.

2233F929-A028-533A-BAB6-8B6EFC960F1F

846066

Fig. 3

##### Etymology.

Named after Hainan Province (China) where the type was collected.

##### Type.

China, Hainan Province: Diaoluoshan National Nature Reserve, on diseased leaves of bamboo, 23 June 2021, R.Y. Liu, holotype HSAUP 1681, ex-type living culture SAUCC 1681.

##### Description.

***Asexual morph***: On WA, hyphae 1.2–3.4 μm diam., hyaline, branched, septate. Conidiophores cylindrical, septate, verrucose, flexuous, sometimes reduced to conidiogenous cells. Conidiogenous cells globose to subglobose, erect, blastic, aggregated in clusters on hyphae, hyaline to pale brown, smooth, branched, 6.4–8.8 × 5.2–7.1 μm, mean ± SD: 7.9 ± 1.1 × 6.1 ± 0.9 μm (n = 40). Conidia globose, subglobose to lenticular, with a longitudinal germ slit, occasionally elongated to ellipsoidal, brown to dark brown, smooth to finely roughened, 5.5–8.5 × 5.0–7.5 μm, mean ± SD: 6.8 ± 0.9 × 6.7 ± 0.7 μm, L/W = 1.0–1.1 (n = 40). ***Sexual morph***: Undetermined.

##### Culture characteristics.

Colonies on PDA flat with entire margin, aerial mycelium white to grey, floccose cottony; reverse white to pale honey colored. PDA attaining 77.5–85.5 mm in diameter after 7 days at 25 °C, growth rate 10.5–12.5 mm/day.

**Figure 3.**
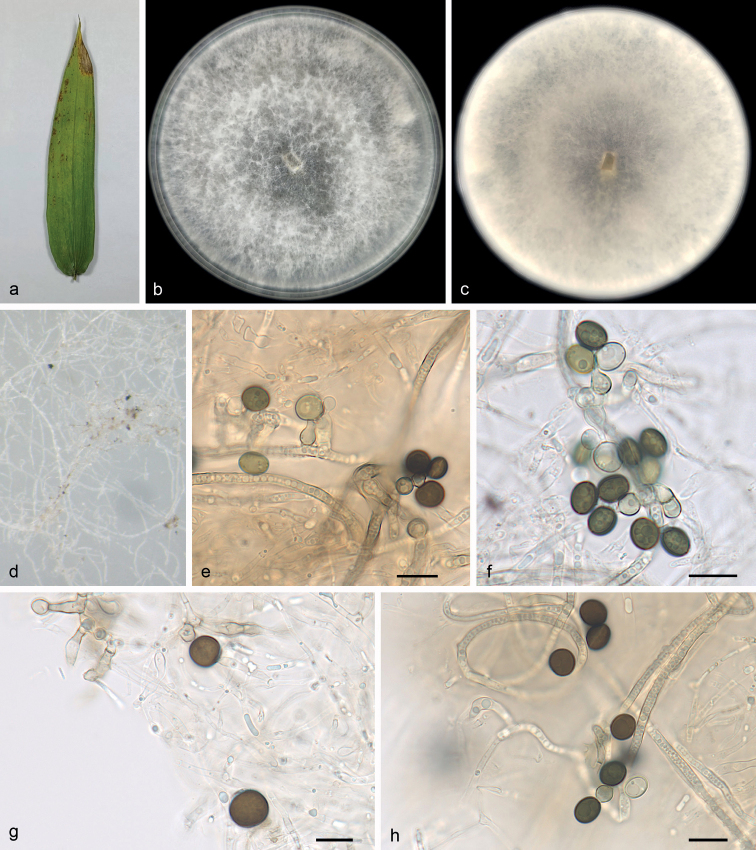
*Apiosporahainanensis* (SAUCC 1681, ex-holotype culture) **a** leaf of host plant **b, c** surface (**b**) and reverse (**c**) sides of colony after incubation for 7 days on PDA**d** conidiomata formed in culture **e, f** conidiogenous cells and conidia **g, h** conidia. Scale bars: 10 μm (**e–h**).

##### Additional specimen examined.

China, Hainan Province: Diaoluoshan National Nature Reserve, on diseased leaves of bamboo, 23 June 2021, R.Y. Liu, paratype HSAUP 1682, ex-paratype living culture SAUCC 1682.

##### Notes.

The two strains (SAUCC 1681 and SAUCC 1682) of *A.hainanensis* clustered together with significant support in an isolated branch basal to *A.sacchari* and related species of the phaeospermum clade (Pintos and Alvarado 2022; Fig. 1). Other species in a more or less similar phylogenetic position include *A.septata* (Y. Feng & Jian K. Liu) X.G. Tian & Tibpromma, *A.piptatheri* (Pintos & P. Alvarado) Pintos & P. Alvarado, *A.longistroma* (D.Q. Dai & K.D. Hyde) Pintos & P. Alvarado, *A.pseudospegazzinii* (Crous) Pintos & Alvarado and *A.fermenti* (S.L. Kwon, S. Jang & J.J. Kim) S.L. Kwon & J.J. Kim. Morphologically, it differs from *A.septata*, *A.piptatheri*, *A.longistroma*, *A.pseudospegazzinii* and *A.fermenti* in its conidia (globose, subglobose to lenticular, 5.5–8.5 × 5.0–7.5 μm in *A.hainanensis* vs. surface view globose to ellipsoid, 8–13 μm long and side view lenticular, 8–14 μm long in *A.septata*, globose to ellipsoidal, 6–8 × 3–5 μm in *A.piptatheri*, asexual morph undetermined in *A.longistroma*, surface view globose, 7–9 μm diam. and side view lenticular, 5–6 μm diam. in *A.pseudospegazzinii*, surface view globose to elongate ellipsoid, 7.5–9 × 7–9 μm and side view lenticular, 6–7 μm diam. in *A.fermenti*; Crous and Groenewald 2013; Dai et al. 2016; Pintos et al. 2019; Feng et al. 2021; Kwon et al. 2021, 2022; Pintos and Alvarado 2021; Tian et al. 2021).

#### ﻿ Apiospora pseudosinensis

Taxon classificationFungiXylarialesApiosporaceae

(Crous) Pintos & P. Alvarado, Fungal Systematics and Evolution 7: 207. (2021)

EB540E35-CA14-55C4-9F0F-D560117D8486

Fig. 4

≡ Arthriniumpseudosinense Crous, in Crous & Groenewald, IMA Fungus 4(1): 148 (2013).

##### Description.

***Asexual morph***: On WA, hyphae 1.1–2.9 μm diam., hyaline, branched, septate. Conidiophores cylindrical, septate, verrucose, flexuous, sometimes reduced to conidiogenous cells. Conidiogenous cells globose to subglobose, erect, blastic, aggregated in clusters on hyphae, hyaline to pale brown, smooth, branched, 9.4–11.0 × 6.1–8.8 μm, mean ± SD: 10.4 ± 0.7 × 7.7 ± 1.1 μm (n = 40). Conidia globose, subglobose to lenticular, with a longitudinal germ slit, occasionally elongated to ellipsoidal, brown to dark brown, smooth to finely roughened, 7.5–11.5 × 7.0–9.5 μm, mean ± SD: 10.1 ± 1.3 × 8.3 ± 0.6 μm, L/W = 1.1–1.3 (n = 40). ***Sexual morph***: Undetermined.

##### Culture characteristics.

Colonies on PDA flat with irregular margin, aerial mycelium white to pale yellow, floccose cottony; reverse pale yellow to yellow. PDA attaining 69.5–78.5 mm in diameter after 7 days at 25 °C, growth rate 9.5–11.5 mm/day.

**Figure 4.**
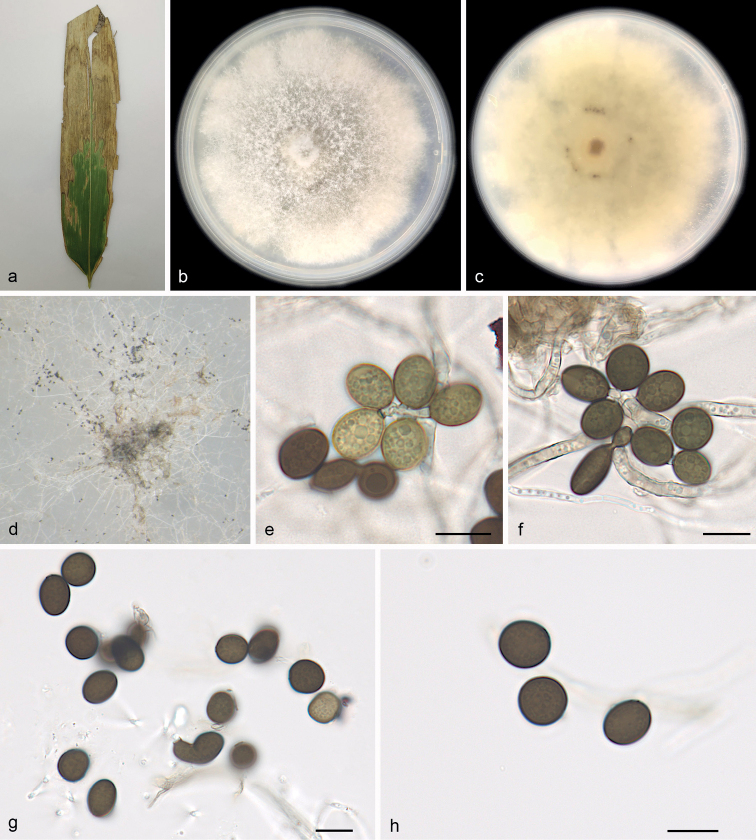
*Apiosporapseudosinensis* (SAUCC 0221) **a** leaf of host plant **b, c** surface (**b**) and reverse (**c**) sides of colony after incubation for 7 days on PDA**d** conidiomata formed in culture **e, f** conidiogenous cells and conidia **g, h** conidia. Scale bars: 10 μm (**e–h**).

##### Specimens examined.

China, Shandong Province: Dongying Botanical Garden, on diseased leaves of bamboo, 15 July 2022, R.Y. Liu, HSAUP 0221, living culture SAUCC 0221; China, Hainan Province: Diaoluoshan National Nature Reserve, on diseased leaves of bamboo, 29 June 2021, R.Y. Liu, HSAUP 0022, living culture SAUCC 0022.

##### Notes.

*Apiosporapseudosinensis* was originally described from bamboo leaves collected in the Utrecht Botanical Garden of the Netherlands (Crous and Groenewald 2013; Pintos and Alvarado 2021). In the present study, DNA sequences obtained from two strains (SAUCC 0221 and SAUCC 0222) collected also from bamboo leaves, were not significantly different from those of *A.pseudosinensis* (Fig. 1). Morphologically, our strains were similar to the original description (conidia 8–10 × 7–10 μm diam. in surface view, 7–8 μm diam. in side view). We therefore consider the newly found strains as *A.pseudosinensis* (Crous and Groenewald 2013; Pintos and Alvarado 2021).

## ﻿Discussion

The family Apiosporaceae was proposed to accommodate genera with apiosporous hyaline ascospores and a basauxic, *Arthrinium*-like conidiogenesis (Hyde et al. 1998). Crous and Groenewald (2013) synonymized *Apiospora* with *Arthrinium* on the basis of the one fungus-one name policy (Hawksworth et al. 2011). Crous and Groenewald (2013) also resolved the genetic identity of multiple species of *Arthrinium* (= *Apiospora*), analysing ex-type collections, and confirmed that most species occur in Poaceae (R.Br.) Barnh. hosts, although some were known from many other plant host families. However, with the aid of additional genetic data from the type species of *Arthrinium*, *Ar.caricicola*, *Apiospora* and *Arthrinium* were separated again as two distinct genera (Pintos and Alvarado 2021). *Arthrinium* species have variously shaped conidia and inhabit Cyperaceae and Juncaceae in temperate, cold or alpine habitats. Most *Apiospora* species have rounded/lenticular conidia and inhabit mainly Poaceae (and many other host plant families) in a wide range of habitats, including tropical and subtropical regions (Pintos and Alvarado 2021; Samarakoon et al. 2022). An epitype for the type species of *Apiospora*, *A.montagnei*, was recently proposed by Pintos and Alvarado (2022).

There are many *Apiospora* species found on bamboos across the world (Table 2). Bamboos (Poaceae) are distributed in tropical and subtropical to mild temperate regions, with the heaviest concentration and largest number of species in China. Due to their abundance and economic importance, it is of great significance to study and identify the fungi growing on bamboo (Feng et al. 2021). In the present study, two new species (*Apiosporadongyingensis* and *A.hainanensis*) are introduced, and another one (*A.pseudosinensis*) is reported for the first time in China. All of them were collected from bamboo leaves and described based on their phylogenetic data and morphological characters. The descriptions and molecular data for species of *Apiospora* represent an important resource for understanding the diversity of bamboo fungi.

## Supplementary Material

XML Treatment for Apiospora dongyingensis

XML Treatment for Apiospora hainanensis

XML Treatment for Apiospora pseudosinensis

## Supplementary materials

Supplementary material 1 Morphological and phylogenetic analyses reveal two new species and a new record of *Apiospora* (Amphisphaeriales, Apiosporaceae) in China Data type phylogenetic Explanation note The combined ITS, LSU, *tef1* and *tub2* genes. https://binary.pensoft.net/file/802183This dataset is made available under the Open Database License (http://opendatacommons.org/licenses/odbl/1.0/). The Open Database License (ODbL) is a license agreement intended to allow users to freely share, modify, and use this Dataset while maintaining this same freedom for others, provided that the original source and author(s) are credited.Rongyu Liu, Duhua Li, Zhaoxue Zhang, Shubin Liu, Xinye Liu, Yixin Wang, Heng Zhao, Xiaoyong Liu, Xiuguo Zhang, Jiwen Xia, Yujiao Wang
